# Overview of the main methods used for estimating catastrophic health expenditure

**DOI:** 10.1186/s12962-023-00457-5

**Published:** 2023-08-08

**Authors:** Huyen Anh Nguyen, Sayem Ahmed, Hugo C. Turner

**Affiliations:** 1 Oxford University Clinical Research Unit, Centre for Tropical Medicine, Ho Chi Minh City, Vietnam; 2 Health Economics and Health Technology Assessment, School of Health and Wellbeing, University of Glasgow, Glasgow, UK; 3 MRC Centre for Global Infectious Disease Analysis, School of Public Health, Imperial College London, Norfolk Place, London, UK

**Keywords:** Catastrophic health expenditure, Health economics, Health financing, Health inequity, Health system capacity, Out-of-pocket

## Abstract

Out-of-pocket payments are expenditures borne directly by an individual/household for health services that are not reimbursed by any third-party. Households can experience financial hardship when the burden of such out-of-pocket payments is significant. This financial hardship is commonly measured using the “catastrophic health expenditure” (CHE) metric. CHE has been applied as an indicator in several health sectors and health policies. However, despite its importance, the methods used to measure the incidence of CHE vary across different studies and the terminology used can be inconsistent. In this paper, we introduce and raise awareness of the main approaches used to calculate CHE and discuss critical areas of methodological variation in a global health context. We outline the key features, foundation and differences between the two main methods used for estimating CHE: the budget share and the capacity-to-pay approach. We discuss key sources of variation within CHE calculation and using data from Ethiopia as a case study, illustrate how different approaches can lead to notably different CHE estimates. This variation could lead to challenges when decisionmakers and policymakers need to compare different studies' CHE estimates. This overview is intended to better understand how to interpret and compare CHE estimates and the potential variation across different studies.

**Supplementary Information:**

The online version contains supplementary material available at 10.1186/s12962-023-00457-5.

## Introduction

Around US$7.5 trillion was spent on health in 2016, accounting for nearly 10% of the global Gross Domestic Product (GDP) [1]. Health expenditure is shared across many different sources and stakeholders, including governmental health spending, non-government funding, health insurance funding and patient out-of-pocket (OOP) payments. OOP payments are one of the most critical healthcare funding sources, accounting for over 50% of the total health expenditure in many low and middle-income countries [1]. OOP payments are expenditures borne directly by an individual/household for health services that are not reimbursed by any third-party (such as a health insurance program) [2, 3]. Households can experience financial hardship when the burden of such OOP payments is significant in relation to their ability to pay. This financial hardship is commonly measured using the “catastrophic health expenditure” (CHE) metric, where the OOP payments exceed certain defined levels [4, 5]. Patients confronted with CHE are generally from low-income households, especially vulnerable groups [5]. Over 100 million people are estimated to be pushed into poverty due to OOP payments every year [6].

CHE has been applied as an indicator in several different areas of the health sector. For example, CHE estimates can be stratified by socioeconomic characteristics to identify potential inequalities from OOP payments [7]. This is a valuable indicator to evaluate the performance of financial protection functions within health systems and the metric is used to monitor progress towards Universal Health Coverage and the Sustainable Development Goals (SDG) [8]. Recently, CHE has had an increasing role within health policy assessments [9, 10]. Averted CHE can be used as a consideration for policymakers when evaluating different healthcare policies/interventions [11].

Despite the importance of this metric, the methods used to measure the incidence of CHE vary across different studies [12] and the terminology used can be inconsistent. This variation in CHE calculations and terms could lead to challenges when policymakers need to compare different studies/estimates [8].

There is an increasing body of literature on the CHE metric (several key papers are highlighted in Box 1). However, there is currently a gap in the literature regarding an overview of the different methods used to measure the incidence of CHE targeted to a public health professional audience. The aim of this paper is to provide an overview of the main approaches used to calculate CHE (including their specific calculations and foundation) and discuss critical areas of methodological variation in a global health context. We also use data from Ethiopia as a case study to explore how different approaches may impact CHE estimates. This is intended to lead to a better understanding of how public health professionals should interpret and compare CHE estimates, and potentially reduce variation across different studies.

Box 1: Key papers related to catastrophic health expenditure calculations

1. Xu, Ke, et al. "Household catastrophic health expenditure: a multicountry analysis." *The lancet* 362.9378 (2003): 111–117.
2. Xu, Ke, et al. "Designing health financing systems to reduce catastrophic health expenditure." (2005).
3. Xu, Ke, et al. "Protecting households from catastrophic health spending." *Health affairs* 26.4 (2007): 972–983.
4. Wagstaff, Adam, and Eddy van Doorslaer. "Catastrophe and impoverishment in paying for health care: with applications to Vietnam 1993–1998." *Health economics* 12.11 (2003): 921–933.
5. Wagstaff, Adam, et al. "Progress on catastrophic health spending in 133 countries: a retrospective observational study." *The Lancet Global Health* 6.2 (2018): e169-e179.
6. Wagstaff, Adam. "Measuring catastrophic medical expenditures: reflections on three issues." *Health economics* 28.6 (2019): 765–781.
7. Hsu, Justine, et al. "Measuring financial protection against catastrophic health expenditures: methodological challenges for global monitoring." *International journal for equity in health* 17 (2018): 1–13.
8. Cylus, Jonathan, et al. "Catastrophic health spending in Europe: equity and policy implications of different calculation methods." *Bulletin of the World Health Organization* 96.9 (2018): 599.
9. Flores, Gabriela, et al. "Coping with health‐care costs: implications for the measurement of catastrophic expenditures and poverty." *Health economics* 17.12 (2008): 1393–1412.

## Main methods used for measuring catastrophic health expenditure

The two main methods used to measure the incidence of CHE are the budget share approach and capacity-to-pay approach. An overview of the methods is presented in Tables 1 and 2. In addition, a glossary of key terms related to CHE is presented in Additional file 1: Table S1.

**Table 1:** Overview of the two main approaches used to measure catastrophic health expenditure

	Budget share approach	Capacity-to-pay approach		
Definition	Catastrophic health expenditure is defined as occurring when out-of-pocket payments exceed a defined proportion of a household’s total income or expenditure	Catastrophic health expenditure is defined as occurring when out-of-pocket payments exceed a defined proportion of a household’s capacity to pay (the available household expenditure remaining after their basic needs have been met)		
Calculation	$\frac{Out\,of \,pocket\,payments}{Total expenditure\,or \,income}>Threshold$	$\frac{Out\,of\,pocket\,payments}{Total\,expenditure-basic\,needs}>Threshold$		
Numerator	Out-of-pocket payments	Out-of-pocket payments		
Denominator	Total household expenditure or total household income	Sub-method 1: Total household expenditure minus actual food spending	Sub-method 2: Total household expenditure minus a standard amount representing subsistence food spending^a^	Sub-method 3: Total household expenditure minus a standard amount representing subsistence spending on food, rent and utilities
Standard thresholds	10% and 25%	25% and 40%	40%	40%
Examples of application	Sustainable Development Goal (indicator 3.8.2), World Bank	Pan American Health Organization, World Bank	World Health Organization	World Health Organization—European Region
Representative papers	Wagstaff and Doorslaer [13], Wagstaff [12]	Xu et al. [14, 15],		

^a^Except for household’s whose food spending is less than the estimated standard amount, where their actual food spending is used within the calculation instead

**Table 2:** Summary of the advantages and limitations of the budget share approach and capacity-to-pay approach

	Budget share approach	Capacity-to-pay approach
Advantages	Simple to apply and requires less data	Less biased by variation in the purchasing capacity between different income classes Development of various forms of the approach to adapt to different settings Sub-methods that account for household size and composition
Limitations	Does not consider variation in the purchasing capacity amongst different income classes Does not typically account for how household size and composition effects household. spending/income and leads to different degrees of economic capacities	Requires more data and therefore can be challenging to apply in LMICs Argued that it does not reflect the impact of out-of-pocket payments on the households' ability to purchase non-medical necessities Uncertainty and controversy regarding what constitutes as “basic needs”

### The budget share approach

The budget share approach (or basic approach) was popularized by researchers from the World Bank [4, 13]. The budget share approach defines CHE as occurring when OOP payments exceed a defined proportion (generally 10% or 25%) of a household’s total income or expenditure within a given period (Table 1) [12, 16–18]. This method supposes that the household budget is always available for healthcare spending. Consequently, it assumes that if healthcare spending is large enough, the household budget will sacrifice another section of necessary spending, such as food, housing and education. The budget share approach is used within the SDG (indicator number 3.8.2) [19].

An advantage of the budget share approach is that it is simple to apply as there is no need to distinguish different types of spending, such as discretionary spending (that can be reduced if necessary) and non-discretionary spending (which cannot be reduced).

The budget share approach has limitations, for example, it does not account for differences in purchasing capacity between different income classes [20]. Notably, people with a lower socioeconomic status will have to spend a larger proportion of their income to meet their basic needs (such as food) compared to richer individuals [8, 16]. Consequently, even when spending the same budget share on healthcare, the poorer groups of a population will have a greater financial hardship compared to richer groups [16, 21]. The budget share approach does not account for this and applying the same threshold to all households regardless of their wealth/income status can underestimate the financial hardship for poorer groups and overestimate it for richer groups of a population [8, 16]. The budget share approach can involve either individual or household weighting depending on what is being reported [16, 17]. However, the approach does not typically account for how the different sizes and composition (i.e. number of adults and children) of households, affects household spending/income and leads to different degrees of economic capacities [13, 16, 22].

### Capacity-to-pay approach

The “capacity-to-pay” (sometimes referred to as the “ability-to-pay”) approach was popularized by Xu et al. [14, 15]. In contrast to the budget share approach, this method assumes that households must first cover their basic needs (Additional file 1: Table S1) before covering healthcare spending [5, 14, 15, 23]. Consequently, this approach assumes that the spending required for basic needs should not be considered part of the resources available for health spending [8], and it is therefore deducted from households’ total expenditure. After this adjustment, the remaining expenditure is the “household’s capacity-to-pay”, which can be associated with healthcare spending and used within the CHE calculation. Different methods are used to approximate expenditure on basic needs, with food spending often used as a proxy [8, 14, 15]. Within this approach, CHE is defined as occurring when OOP payments exceed a defined proportion (generally 40%) of a household’s capacity to pay within a given period (Table 1).

The capacity-to-pay approach has important advantages. As it includes an adjustment for basic needs spending, it could be argued it provides a better measurement of CHE compared with the budget share approach. It is less biased by differences in the purchasing capacity between different income classes and it recognizes that poorer households spend a higher proportion of available resources on essential items than richer household [8]. Furthermore, some of the sub-methods of this approach also have adaptations that use equivalence scales (Additional file 1: Table S1) to account for differences in household structures [14].

A limitation of the capacity-to-pay approach is that it requires more data and is therefore not as simple to implement as the budget share approach. This is particularly important for low and middle-income country settings where typically less data are available. Additionally, as the capacity-to-pay approach excludes basic needs from the households’ total expenditure, it has been argued that it cannot reflect how the OOP payments impact the households' ability to buy non-medical necessities [24]. In addition, there is uncertainty and controversy regarding what constitutes “basic needs”. Although food spending is often used as a proxy for basic needs it may not always be adequate, particularly in some high- and middle-income countries where households have to spend a larger proportion of their expenditure on housing and utilities [16, 25]. For example, heating is an important basic need in many colder countries. This has led to the development of different forms of the capacity-to-pay approach that calculate basic needs differently to adapt the approach for different settings [16] (see Table 1):

1. Basic needs reflected by actual food spending: Within this sub-method, the basic needs adjustment is only based on the household’s actual food spending (i.e. actual food spending is deducted from the household’s total expenditure with no other adjustments). As stated, it is debatable whether food is an adequate proxy for basic needs and this does not recognize that some food spending is discretionary [8]. In addition, this method does not account for how the household’s actual food spending may be influenced by their OOP payments [26]. For example, if a household was spending less on food because of their spending on healthcare, it would appear to have a greater capacity to pay and would therefore be less likely to be counted as a experiencing CHE relative to a household which was spending more on food [26]. This sub-method is used by the Pan American Health Organization and World Bank [12, 27].
2. Basic needs reflected a standard amount representing basic spending on food (partial normative food-spending method): Within this sub-method, the basic needs adjustment is also based on food spending but it addresses the limitations of the actual food-spending sub-method by deducting a standard amount of food spending from each household’s total expenditure rather than using actual food spending [14]. This standard amount is an estimate of the amount of money a household requires to meet its basic food needs (i.e. it tries to only reflect non-discretionary food spending) [28] and aims to arrive at a standard expenditure level representing basic needs. This is approximated based on average food spending per equivalent person of households whose food share of their total household expenditure is between the 45^th^ and 55^th^ percentiles of the sample [14]. This sub-method can use equivalence scales to account for differences in household size and composition. When a household’s food spending is less than this estimated standard amount, their actual food spending is used within the calculation instead. Due to this, households whose food spending is just above or below the standard amount are treated differently [8]. This sub-method is referred to within the WHO guidelines for CHE calculation [5].
3. Basic needs reflected a standard amount representing basic spending on food, housing and utilities (normative spending on food, housing and utilities method): This sub-method extends the concept of basic needs to include spending on housing and utilities (water, gas, electricity and heating), in addition to the cost of food. Within this sub-method, a standard amount that reflects basic spending on food, housing and utilities is deducted from all the households' total expenditures. This estimated standard amount of basic spending is based on the average spending on food, rent and utilities among households between the 25th and 35th percentiles of the sample ranked by total household expenditure per equivalent person i.e. the standard amount is based on the spending from among relatively poor households [14]. This sub-method can also use equivalence scales to account for differences in household size and composition. Households with total expenditures below this estimated standard amount of basic spending are considered to be catastrophic spenders if they incur any OOP payments (and the method allows a household to have negative capacity-to-pay) [14]. This sub-method was developed and used by the WHO Regional Office for Europe [16].

## Sources of variation in the calculation of catastrophic health expenditure

In the previous section, we described the two main approaches used to measure CHE. It is important to note that the two different approaches and the exact method/assumptions used for the CHE calculations can significantly impact the output. Using data from Ethiopia as a case study (see Box 2) we show how different thresholds and methods can result in notably different CHE estimates. Within this case study the estimated minimum OOP payment that would result in CHE varied between US$111 and US$337, depending on the assumptions/approach. Although this is a crude case study, it highlights the potential variation in CHE calculations.

Box 2: Case study relating to Ethiopia Ethiopia is classed as a low-income country with a per capita income of around US$850 in 2019 [29]. According to the 2015/16 Ethiopian National Health Accounts (NHA) [30], OOP payments represented 83% of the total healthcare spending in the country. Due to the high level of OOP payments, it is important to understand at what level they could be leading to CHE.

The table below utilizes data from Ethiopia’s NHA [30] and shows the different levels at which OOP payments would result in CHE according to the different approaches and thresholds that are commonly employed. Although this crude example uses averages for the data in the denominator rather than individual level data, it does indicate the magnitude of the differences between the approaches.

**Table:** 

Approach	Threshold (%)	Denominator	The out-of-pocket payment that results in CHE	
			Ethiopian birr (ETB)	US$^a^
Budget share	10	Household income	2423	111
Budget share	10	Household expenditure	2931	135
Capacity-to-pay^b^	25	Expenditure minus actual food spending	3200	147
Capacity-to-pay^b^	40	Expenditure minus actual food spending	5121	236
Budget share	25	Household income	6057	279
Budget share	25	Household expenditure	7328	337

These calculations are based on an average food spending of ETB16,508, a household income of ETB24,227 and a household expenditure of ETB29,310 [30].

^a^Using the 2016 exchange rate (US$1 = ETB21.73) [31].

^b^Capacity-to-pay approach was applied using sub-method 1 (Table 1), where the basic needs was represented by actual food spending.

Table 1 illustrates the basic calculation used to estimate the incidence of CHE, and how it is influenced by three factors: the denominator, numerator and threshold. In the following sections, we outline the sources of variation impacting these factors. Firstly, we discuss the issues related to the denominator of the calculation. We then examine the numerator (i.e. the estimation of the out of pocket payment). Finally, we discuss the use of different thresholds within CHE calculations.

### The denominator: household expenditure vs income

The budget share approach uses either total household income or total household expenditure as the denominator within the CHE calculation. Importantly which one is used can influence the result (Box 2). For example, a report related to CHE estimates in Malaysia [32] found that with the range of threshold values investigated (5–40%), the estimated proportion of households facing CHE varied between 0.04–9.9% when using household expenditure within the denominator, compared to 0.03–5.5% when using income.

Although both were used extensively as the denominator in earlier studies [13, 33–35], household expenditure is now more frequently used within the budget share approach [12]. However, the use of income as the denominator is sometimes applied for high-income settings and when there is no household expenditure data [12]. Therefore, the choice between using income or household expenditure within the budget share approach can depend on the setting and available data. Wagstaff [24] highlighted that whether household expenditure or income provides the more reliable denominator depends on the setting; with household expenditure providing the more reliable denominator in settings where households need to borrow to finance the OOP payments and income where they do not. This suggests that an income-based denominator may be more relevant to high-income countries and a expenditure-based denominator more relevant to low and middle-income country settings.

In contrast, the capacity-to-pay approach does not use income and uses total household expenditure within the denominator, adjusting it for spending on basic needs; but there is variation in what is used as the proxy for “basic needs” spending. Further work would be beneficial to improve how “basic needs” is defined with the capacity approach calculations (potentially more in line with other theoretical frameworks).

It is also important to consider how the household expenditure or income data are being collected. Household expenditure is often collected through household expenditure surveys [36]. However, the way the survey is designed can influence how respondents answer questions related to expenditure. For example, the use of a face-to-face versus a self-report questionnaire, the sampling strategy, and the recall period can all affect the expenditure data quality. There are also several issues associated with estimating/collecting data on household income, especially in low-and-middle income countries where a large proportion of the population depends on an informal economy [37]. For example, there can be a high rate of non-response to questions regarding income in household surveys [38]. Reporting income can also be challenging for those that are self-employed, particularly for subsistence farmers and those that receive seasonal income, which is common in low-income settings [12].

Consequently, the choice of measure for the denominator and its associated measurement factors need to be considered when comparing CHE estimates.

### The numerator: what is included as an out-of-pocket payment and how is it calculated?

OOP payments are the numerator in the CHE calculation for both the budget share and the capacity-to-pay approaches. However, there is limited information regarding how OOP payments are measured. WHO has claimed that information collected on OOP payments is less reliable than other sources of health spending [39].

There is variation in the breakdown and the components/cost types included in the OOP payments (Table 3) [40]. All studies would consider non-reimbursed payments related to direct medical costs, i.e. the costs directly associated with the use of medical services/resource (such payments for medicines) and these are a key driver of health expenditure. However, some studies also consider certain types of direct non-medical costs incurred by patients, i.e. costs related to non-medical resources (such as costs related to travel) (Table 3). In addition, OOP payments could also include informal payments, such as for traditional medicine (see [41] for the definition from WHO) [40]. These informal payments are a significant challenge to estimate since they can be sensitive information and depend on the context of each setting [42]. Importantly, the main cost drivers in OOP payments vary across different settings (Table 3).

**Table 3:** Examples of variation regarding the breakdown of the components included within out-of-pocket payment calculations from low and middle-income countries case studies

Country and year	Out-of-pocket payments components	
	Category	Proportion (%)
Sri Lanka [43] 2009	Fees to private medical practitioners	53.3
	Medical and pharmaceutical products	33.6
	Other	13.1
Maldives [43] 2012	Medical and pharmaceutical products	62.4
	Outpatient care	35.6
	Inpatient care	1.7
	Other	0.3
Ethiopia [30] 2017	Medical and pharmaceutical products	45.0
	Medical services	26.0
	Bed and accommodation	12.0
	Travel	10.0
	Other	7.0
Myanmar [44] 2015	Medical and pharmaceutical products	35.3
	Outpatient care	43.4
	Inpatient care	19.4
	Travel	1.6
	Other	0.3

There are currently limited standardized tools for OOP payment data collection [5, 45]. OOP payments can also be taken from household expenditure/living standard surveys. However, it is important to note that these surveys are not always designed to collect OOP payment information (as they are usually designed to measure living standards or overall household expenditure). It has been highlighted that effective instruments specialized in collecting OOP payments should be developed [45]. A further issue with OOP payment data is that it is challenging to track whether the money will be reimbursed or not by a health insurance program later, potentially leading to an overestimate of OOP payments [36]. As a solution to this, it has been proposed that OOP payments should be reported from both the patients’ and providers’ sides [39]. However, this would be more labour intensive.

Differences in how OOP payments have been calculated (including what cost types have been included and how it was collected) need to be considered when comparing different CHE estimates. It would be beneficial if similar reporting frameworks to those often applied within economic evaluations (such as cost-effectiveness analysis) regarding clarifying what types of costs are included were considered for CHE calculations.

### The thresholds for CHE estimation

A threshold that represents the point above which OOP payments result in financial hardship to the household is needed within CHE calculations. The threshold is set at different values for the two different approaches and it is important to consider what specific thresholds are being used when comparing CHE estimates.

The budget share approach uses thresholds of 10% or 25% of total household expenditure or income. The 10% threshold is more commonly applied than the 25% threshold [12]. In contrast, within the capacity-to-pay approach, a threshold of 40% of the household capacity to pay is recommended [14], although some studies have recently applied a threshold of 25% for the basic needs when using the actual food-spending sub-method [12].

The case study in Box 2 illustrates the significant impact the different thresholds can have on the levels at which OOP payments would result in CHE. To handle the uncertainty regarding which threshold to use, it is common for studies to report the results using multiple thresholds. There are some cases where the threshold is changed in line with the socioeconomic status of the study setting [46, 47]. The use of different thresholds is important as it allows the investigation of inequity issues from different points of view/different societal values.

## Further considerations

Both the budget share and capacity-to-pay approaches do not typically consider the sources of household OOP payments. Therefore, they do not account for the variation in the capacity of different households to cope with OOP payments, such as differences in their level of savings, other assets, credit or ability to borrow money from friends/relatives [48]. In addition, they do not typically capture the lifetime consequences of OOP payments and the negative long-term impact of the coping strategies needed to finance them [24].

Beyond the specific calculation approaches, there are also general limitations of the CHE metric that are important to be aware of. For example, it does not capture how OOP payments could create a financial barrier for patients accessing healthcare. High OOP payments could lead to socioeconomically disadvantaged patients being less likely to use health services [49] which can lead to a vicious cycle of poor health and poverty. In addition, the incidence of CHE does not show the extent to which the OOP payments cause hardship or the resulting level of impoverishment. The CHE metric also does not capture the patients' burden beyond OOP payments, such as from the patients lost productivity [50].

To address these limitations of typical CHE calculations, other methods have been developed [8, 24, 33]. For example, Flores et al*.* [33] developed a calculation that considers healthcare spending stratified by the financial source, including income, savings, borrowing, sales of assets and proposed a coping‐adjusted catastrophic payment ratio [33]. CHE calculations can also be modified to capture the lifetime consequences of OOP spending [24].

In addition, other metrics can be used in this area. For example, impoverishment calculations can be used in addition to the CHE metric to show how far people are pushed below the poverty line as a result of healthcare spending, as well as how healthcare spending may push people who are already poor even further into poverty [51], giving an indicator of the resulting level of impoverishment or intensity of the OOP expenditure. The catastrophic payment overshoot indicator can also be used to measure the percentage by which OOP expenditure of the household exceeds the threshold of reference [35, 37]. Furthermore, a new metric known as “catastrophic costs” has more recently emerged in tuberculosis studies [34]. Compared to CHE, “catastrophic costs” is a broader metric, covering both direct costs and productivity costs (indirect costs). Catastrophic costs are typically defined as occurring when the total costs (including both direct and productivity costs) for healthcare exceed 20% of the annual household income [34]. This metric can more fully capture the burden on the household beyond the OOP payments.

To improve CHE measurement, further research to understand the sequence of choices individuals make concerning health care and other needs is needed (for example, can individuals not pay for health care because they have already paid for their basic needs or vice versa).

## Conclusion

The budget share and the capacity-to-pay approaches are the two main methods used for estimating CHE and are applied widely in the healthcare sector. Although these approaches appear similar there are important differences in their calculations as well as their foundation (Table 1). It is important to note that the two different approaches, as well as the exact method/assumptions used within the CHE calculations, can have a significant impact on the output (as highlighted in our case study in Box 2). This variation in CHE calculation could lead to challenges when decision makers and policymakers need to compare different studies/estimates. It is therefore important that the methodology associated with calculations is clearly reported. This highlights the importance that studies in this area clearly report what approach is being used, the specific assumptions of the calculation and outline the corresponding advantages and disadvantages. It is also noteworthy that although CHE is a useful measure, it does not capture the patient’s full financial burden. Depending on the context, other measures and calculations may also need to be considered.

### Supplementary Information

**Additional file 1: Table S1**. Glossary of key terms related to catastrophic health expenditure.

## Data Availability

All data generated or analysed during study are included in this article.
