# Supplementary material for: Overview of the main methods used for estimating catastrophic health expenditure
Source: Cost Eff Resour Alloc. 2023 Aug 8;21:50. doi: 10.1186/s12962-023-00457-5 (PMC10408045; doi:10.1186/s12962-023-00457-5)
Supplement: Supplementary file 1 — Additional file 1: Table S1. Glossary of key terms related to catastrophic health expenditure. [file 12962_2023_457_MOESM1_ESM.docx]

**Supporting Table 1: Glossary of key terms related to catastrophic health expenditure**

| **General terms** | |
| --- | --- |
| **Catastrophic health expenditure**  **(or catastrophic out-of-pocket expenditure or catastrophic health spending)** | A metric that captures when out-of-pocket payments for healthcare services goes above a defined threshold of household income or expenditure, indicating it is resulting in financial hardship for the household. |
| **Out-of-pocket payments** | Out-of-pocket payments are expenditures borne directly by an individual/household for health services that are not reimbursed by any third-party, such as a health insurance program. In practice, there can be variations in the precise definition of out-of-pocket payments and what types of cost components are included within it. |
| **Equivalence scales (or household equivalence scales)** | Equivalence scales can be used to adjust household consumption data to an individual level considering the size and age composition of the household and the economies of scale surrounding household consumption. |
| **The budget share approach** | |
| **The budget share (or the basic) approach** | An approach used to measure the incidence of catastrophic health expenditure. It defines catastrophic health expenditure as occurring when out-of-pocket payments exceed a defined proportion of a household’s total income or expenditure within a given period. |
| **The capacity-to-pay approach** | |
| **The capacity-to-pay (or ability to pay) approach** | An approach used to measure the incidence of catastrophic health expenditure. It defines catastrophic health expenditure as occurring when out-of-pocket payments exceed a defined proportion of a household’s capacity to pay. |
| **Household’s capacity to pay** | The total household expenditure remaining after their basic needs have been met. |
| **Basic needs (or basic subsistence need, or standard amount)** | The assumed amount needed for a household to maintain its basic needs. Different methods are used to approximate basic needs (Table 1), with food spending commonly used as a proxy. |
